# Lactate and lactylation: metabolic architects of tumor progression and metastasis

**DOI:** 10.1007/s13402-026-01182-w

**Published:** 2026-04-07

**Authors:** Hanyue Ma, Zhuo Gu, Zhenxin Wang

**Affiliations:** 1 Department of Oncology, The First Affiliated Hospital of Soochow University, Suzhou, 215006 China; 2 Department of Surgery, The Second Affiliated Hospital of Soochow University, Suzhou, 215004 China

**Keywords:** Lactate, Lactylation, Metabolic reprogramming, Tumor progression and metastasis, Oncogene

## Abstract

Lactate and its associated modification, lactylation, have emerged as key regulators in influencing various cellular processes. This review explores the multifaceted roles of lactate and lactylation, highlighting their involvement in metabolic reprogramming and the modulation of key signaling pathways. Lactate and lactylation influence cell adhesion, protein degradation, and angiogenesis, contributing to tumor invasion and metastasis. These metabolic alterations maintain cancer stem cell characteristics, support tumorigenesis and resistance to therapy. Lactate and lactylation also facilitate immune evasion in the tumor microenvironment (TME) by modulating immune cell function and immune checkpoint pathways. The complex interplay between lactate, lactylation, and various cellular and immune mechanisms underscores the potential of targeting lactate-related pathways as a therapeutic strategy for cancer treatment. Herein, we provide a comprehensive overview of the current understanding of lactate and lactylation in cancer, offering insights into their roles as key drivers of tumor progression and metastasis.

## Clinical trial number

Not applicable.

## Introduction

Lactate, the end product of glycolysis, has long been recognized as an important metabolite in cellular bioenergetics and metabolic homeostasis across diverse biological contexts. In cancer, the formulation of the Warburg effect revealed that tumor cells exhibit enhanced aerobic glycolysis even in the presence of sufficient oxygen, while retaining functional oxidative phosphorylation, reflecting a high degree of metabolic flexibility. This metabolic configuration leads to substantial lactate accumulation within the tumor microenvironment. Beyond serving as a metabolic intermediate, lactate is now appreciated as an active metabolic fuel and signaling molecule that facilitates intercellular communication through the lactate shuttle. The glycolytic dependency of tumor cells and rapidly proliferating cells positions lactate as a core factor in energy metabolism reprogramming, supplying both the energy and biosynthetic precursors needed for rapid cell proliferation. Nowadays, increasing researches are focusing on how lactate influences tumor biology through various mechanisms. Increasing research efforts have focused on elucidating how lactate-derived signals are transduced into downstream regulatory programs that shape tumor behavior. In 2019, Zhao et al. first identified histone lysine lactylation (Kla) which unveils a novel role of lactate in regulating gene expression and promoting cellular homeostasis [1]. Since this discovery, lactylation has been recognized to extend beyond canonical histone-associated epigenetic regulation, also occurring as a post-translational modification (PTM) on non-histone proteins, including metabolic enzymes and signaling regulators, thereby broadening the spectrum of lactate-responsive cellular processes beyond chromatin regulation. Table 1 highlights the widespread and functionally diverse nature of lactylation across cancer types, including colorectal cancer, breast cancer, lung adenocarcinoma, liver cancer stem cells, gastric cancer, bladder cancer, and prostate cancer. By targeting both chromatin-associated histones and metabolic enzymes, lactylation links lactate accumulation to coordinated changes in gene regulation, metabolic reprogramming, and tumor-promoting phenotypes, underscoring its emerging role as a key metabolic–epigenetic regulator in cancer.

Historically, histone modifications were regarded as a series of PTMs occurring on histones, typically including acetylation, methylation, phosphorylation, ubiquitination, and crotonylation, which collectively fine-tune protein function and cellular signaling. Although many PTMs participate in tumor progression, this review focuses on lactylation because it represents a unique molecular interface between altered tumor metabolism and downstream epigenetic and non-epigenetic regulation. In this context, histone lysine lactylation is discussed as a central mechanistic axis because it provides the most clearly defined and experimentally tractable link between metabolic perturbations and transcriptional reprogramming. At the same time, accumulating evidence indicates that lactylation also occurs on non-histone proteins, including metabolic enzymes and signaling regulators, and these non-histone lactylation events are discussed as complementary regulatory layers throughout this review. Thus, although histone lactylation serves as an organizing backbone for mechanistic integration, the scope of this review encompasses both histone and non-histone lactylation to reflect the broader regulatory landscape.

**Table 1:** The sites and functions of lactylation in different tumors

Diseases	Substrate (Gene/Prtotein)	Lactylation site(s)	Function	Ref.
Colorectal cancer	Histone H3	K18	Promoting the transcription of the RUBCNL (Rubicon-like autophagy enhancer) gene, facilitating autophagosome maturation, enhancing resistance to bevacizumab, and inducing metabolic reprogramming.	[2]
	PFKP	K688	Weakening the metabolic flux of the glycolytic pathway, reducing lactate production through negative feedback, and promoting rapid tumor proliferation Reducing enzyme activity, affecting the efficiency of glycolytic metabolism, and influencing cellular energy metabolism Influencing cell proliferation, differentiation, and metabolism	[3]
	ALDOA	K147		
	Histone H2A.V	K120		
	Histone H3	K14la, K23la		
	Histone H4	K8la, K12la,		
Breast cancer	Histone H3	K18	Promoting tumor cell proliferation, migration, and invasion, and modifying the expression of genes related to cell proliferation, migration, and invasion, such as ZWINT, ECT2, ANLN, and EZR	[4]
Lung adenocarcinoma	Histone H4	K8la, K6la	Regulation of telomerase transcription via Sp1 by modulating histone H4K8 and H4K16 lactylation, leading to telomerase inhibition, telomere dysfunction, and induction of cellular senescence in lung adenocarcinoma.	[5]
Liver cancer stem cells	Histone H3	K56	Maintaining the stem cell characteristics of Liver CSCs	[6]
	ALDOA	K230/322	Regulating the stem cell characteristics of Liver CSCs	
Gastric cancer	Histone H3	K9, K18, K56	Modulating the expression of LDHA through GLUT3, affecting the lactylation levels of histone H3 (H3K9, H3K18, H3K56), influencing the epithelial-mesenchymal transition (EMT) process and promoting the proliferation, migration, and invasion of gastric cancer cells	[7]
	Histone H4	K8, K12		
Bladder cancer	Histone H3	K18	Activating the Hippo signaling pathway through circXRN2 to inhibit H3K18 lactylation, suppressing the proliferation, migration, and glycolytic metabolism of bladder cancer cells, ultimately inhibiting tumor progression	[8]
Prostate caner	Lysine		Enhancing the function of transcriptional enhancers, promoting angiogenesis, and inhibiting anti-angiogenic signals	[9]

**Fig. 1:**
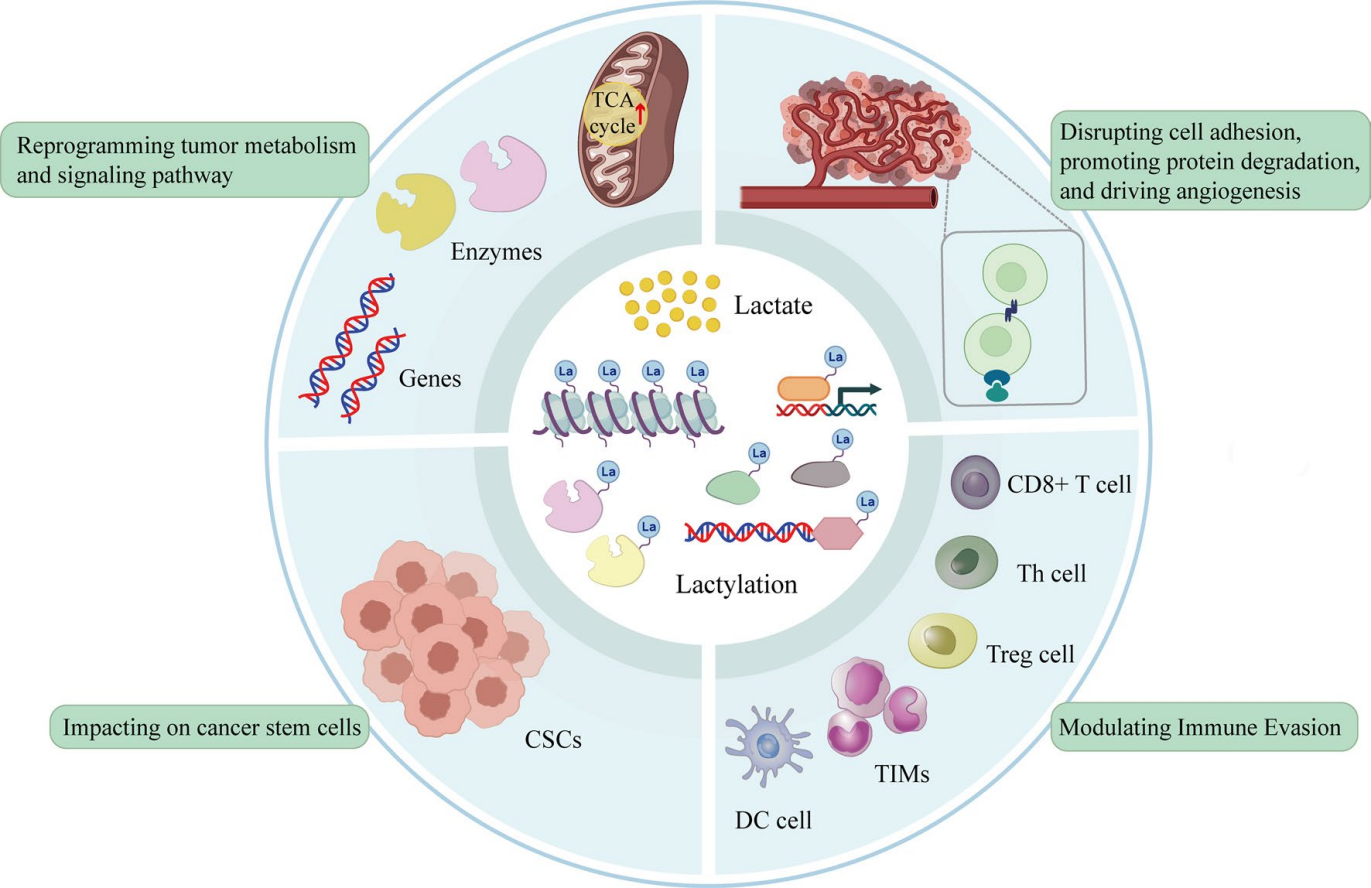
Four key mechanisms by which lactate and lactylation influence tumor progression and metastasis. (**A**) Reprogramming tumor metabolism and signaling pathways. (**B**) Disrupting cell adhesion, promoting protein degradation and driving angiogenesis. (**C**) Impacting on CSCs. (**D**) Modulating immune evasion

Currently, lactylation has been recognized as a covalent modification in which lactate-derived acyl groups are added to specific lysine residues on both histone and non-histone proteins, thereby regulating protein structure, activity, and cellular function through multiple mechanisms. (Fig. 1). Notably, histone Kla primarily operates at the chromatin level to regulate gene transcription and epigenetic states, whereas non-histone lactylation directly modulates metabolic enzymes and signaling proteins, enabling rapid and context-dependent control of protein activity [10, 11]. These functionally complementary regulatory layers integrate metabolic reprogramming with coordinated transcriptional and post-translational control, thereby amplifying lactate-driven tumor regulation. By centering on lactylation as a representative and mechanistically integrative modification, this review seeks to provide a coherent framework for understanding how metabolic cues are translated into coordinated regulatory outputs in cancer.

## The role of lactate and lactylation in reprogramming tumor metabolism and signaling pathway

Metabolic reprogramming, a hallmark of malignancy first identified a century ago, forms the foundation of cancer progression. Lactate and lactylation, although closely related, represent two distinct biological concepts: lactate functions primarily as a metabolic intermediate and signaling molecule, and lactylation acts as a PTM that links cellular metabolism to gene regulation. Recently discovered lactylation regulates gene-driven tumor metabolism and impacts processes ranging from glycolysis to tumor progression.

### Lactate as a metabolic substrate and signaling molecule in tumors

Tumor cells are characterized by an elevated rate of glycolysis, resulting in the accumulation of lactate, which serves as a crucial energy source to support their growth and survival. Lactate is utilized by tumors in two primary ways. On one hand, upon interaction with cancer cells, cancer-associated fibroblasts (CAFs) undergo metabolic reprogramming, exhibiting aerobic glycolysis and producing increased amounts of lactate. This lactate is then taken up by cancer cells where it is further metabolized to support their growth and metastasis [12]. On the other hand, hypoxic cells generate large amounts of lactate via the Warburg effect which is then transported to adjacent well-oxygenated cells through monocarboxylate transporters (MCTs) to fuel oxidative phosphorylation [13]. Considering lactate in the TME exhibits autocrine, paracrine, and endocrine-like functions, these actions not only promote efficient energy redistribution through lactate shuttle mechanisms and ensure an adequate energy supply for tumor cells [14], but also stimulate intercellular signaling via autocrine-paracrine loops [15], enhancing cancer cell invasion and metastasis.

Moreover, accumulating evidence indicates that metabolic remodeling driven by glycolysis and lactate accumulation in the TME also reshapes mitochondrial metabolism in immune cells, thereby indirectly influencing antitumor immunity. In particular, alterations in mitochondrial bioenergetics, redox balance, and metabolite fluxes in dendritic cells have been shown to affect antigen presentation efficiency and T-cell activation, linking tumor-associated metabolic reprogramming to immune modulation within the TME [16]. It has been proved that lactate activates the oncogene c-Myc [17] and increases expression of the glutamine transporter ASCT2 and glutaminase 1 (GLS1) where their activation drives the TCA cycle and establishes a positive feedback loop between glutamine metabolism and lactate production [18], ultimately fueling energy production and promoting tumor cell proliferation and metastatic potential. In breast cancer, c-Myc has been observed to enhance its expression by promoting histone lactylation modifications and upregulating serine/arginine splicing factor 10 (SRSF10) which drives alternative splicing of MDM4 and Bcl-x, thus promoting the proliferation of breast cancer cells. Reducing glycolytic rates through chemotherapy and blocking the c-Myc-SRSF10 axis can effectively limit the progression of breast cancer [19]. In pancreatic cancer, the epigenetic regulator protein arginine methyltransferase 5 (PRMT5) silences the expression of the tumor suppressor F-box/WD repeat-containing protein 7 (FBW7), giving rise to the upregulation of c-Myc and, in turn, promotes aerobic glycolysis and enhances the proliferation of pancreatic cancer cells [20]. It has also been found that homeodomain-interacting protein kinase (HIPK) inhibits the ERK-cMyc axis, leading to a reduction in cMyc protein levels and downregulation of cMyc-targeted glycolytic genes, thereby suppressing the proliferation of pancreatic cancer [21].

Alongside transcriptional and metabolic regulation, targeting mitochondrial dynamics has emerged as a complementary strategy to disrupt tumor metabolic fitness. In gastric cancer models, pharmacological interference with mitochondrial fission and mitophagy has been shown to impair tumor cell survival, migration, and stemness while sparing normal cells, highlighting mitochondria as a vulnerable metabolic node that cooperates with glycolytic and lactate-driven pathways in sustaining tumor progression [22].

### Lactylation as a downstream epigenetic mechanism linking lactate metabolism to gene regulation

Distinct from lactate itself, lactylation represents a metabolite-derived lysine modification that functions as an epigenetic and post-translational regulatory mechanism, directly modulating enzymatic activity and transcriptional programs.

Advancements in high-resolution liquid chromatography coupled with tandem mass spectrometry (LC-MS/MS) have highlighted the pivotal role of histone Kla, independent of the non-specific effects of lactate-induced acidosis. At the chromatin level, histone Kla reshapes transcriptional programs governing central metabolic pathways, including the tricarboxylic acid cycle as well as carbohydrate, amino acid, fatty acid, and nucleotide metabolism. In non-small cell lung cancer (NSCLC) cells, lactate-derived histone lactylation within the promoters of HK-1 and IDH3G enhances the transcriptional activity of Succinate Dehydrogenase Complex Flavoprotein Subunit A (SDHA) and Isocitrate Dehydrogenase 3 Non-Catalytic Subunit Gamma (IDH3G) [23]. Given that SDHA participates in succinate oxidation and IDH3G catalyzes isocitrate decarboxylation, their transcriptional upregulation promotes TCA cycle activity and alters cellular energy metabolism. In parallel with chromatin-based regulation, lactylation also operates at the post-translational level through direct modification of non-histone proteins. Through direct Kla rather than changes in extracellular lactate levels, Kla modification at the K147 site of fructose-bisphosphate aldolase A (ALDOA) led to enzyme inhibition, consequently affecting the glycolytic pathway [24]. Research has further shown that lactylation of specific metabolic enzymes, including Adenylate Kinase 2 (AK2), an adenylate kinase responsible for maintaining intracellular adenine nucleotide homeostasis, promotes the growth of liver cancer cells [25]. These findings establish lactylation as a dual-layer regulatory mechanism that integrates transcriptional control with direct enzymatic modulation.

Additionally, it should be noted that not all lactate-associated transcriptional changes are mediated by lactylation. In prostate cancer cells, lactate itself, acting as a metabolic cue, induces the upregulation of lipid metabolism-related genes (ACLY and SREBF2) and increases histone acetylation levels, particularly at the H3K9 and H3K27 sites [26], thereby enhancing invasive capabilities. This mechanism is driven by lactate signaling rather than histone Kla and is therefore mechanistically distinct from lactylation-dependent regulation.

**Fig. 2:**
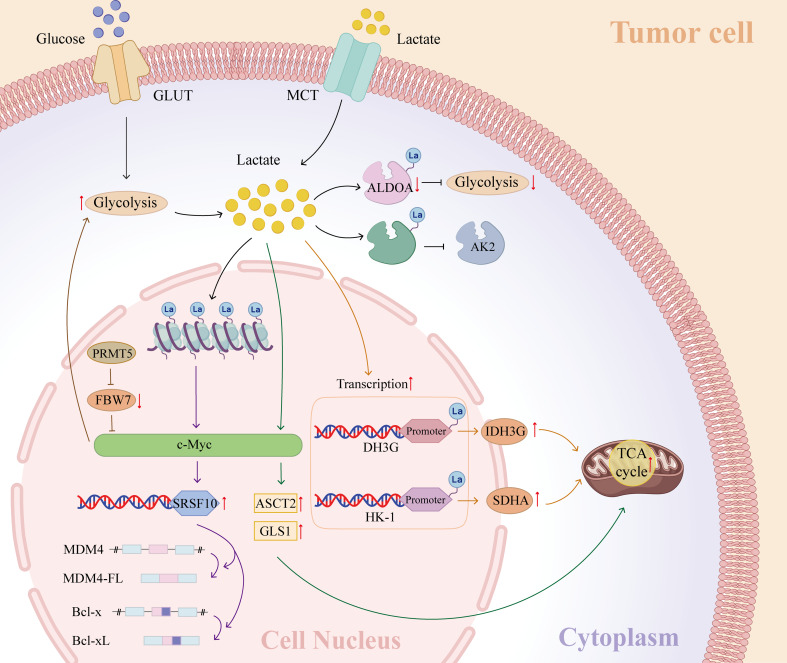
Regulating in tumor metabolism and metastasis via epigenetic and glycolytic pathways. C-Myc boosts its expression via histone lactylation, upregulating SRSF10 to alter MDM4 and Bcl-x splicing. PRMT5 epigenetically suppresses FBW7 expression, while enhancing c-Myc and glycolysis. Lactate activates c-Myc, increasing ASCT2 and GLS1 expression, propelling the TCA cycle for energy. Lactate inhibits AK2 and ALDOA, upregulating ACLY and SREBF2, fostering tumor growth and metastasis

These studies indicate that lactate functions upstream as a metabolic substrate or signaling factor, and lactylation acts downstream as an epigenetic and post-translational mechanism that converts metabolic alterations into coordinated transcriptional and enzymatic regulation, ultimately influencing tumor metabolism and progression (Fig. 2). Therefore, Kla-based targeted therapies represent a promising approach and warrant further investigation into the context-dependent functions of lactylation in tumors.

Beyond histones, non-histone Kla substantially expands the functional landscape of Kla and should be considered a distinct and complementary regulatory axis. Non-histone lysine Kla has been identified on metabolism-related enzymes, where lactylation directly modifies protein function, thereby driving tumor metabolic reprogramming [27, 28]. Systematic proteomic analyses have revealed that non-histone lactylated substrates can be broadly categorized into three functional classes: metabolic enzymes, transcriptional and chromatin-associated regulators, and signaling or stress-response proteins [29]. Within this framework, non-histone lactylation fine-tunes cellular metabolic flux and stress adaptation by directly modulating the activity of key metabolic enzymes, for instance PKM2 [30], G6PD [31] and NMNAT1 [32]. Concurrently, lactylation reshapes transcriptional [33] and epitranscriptomic [34] programmes by targeting gene regulatory factors, thereby coupling metabolic cues to inflammatory signaling, cell fate decisions and immune regulation [35]. By modifying signaling and stress-response proteins, lactylation dynamically governs inflammatory pathways [27], intercellular communication [36] and tissue damage responses [37] under pathological conditions. These observations position non-histone lactylation as a rapid, reversible, and context-dependent regulatory layer that operates in parallel with histone lactylation. Metabolic reprogramming and lactate accumulation act as upstream drivers, enabling lactylation of diverse non-histone substrates and converging on key functional nodes to coordinate metabolic adaptation, gene expression, genome maintenance and immune responses [38]. While histone lactylation predominantly establishes permissive transcriptional states, non-histone lactylation fine-tunes cellular behavior through direct regulation of protein activity, localization and protein–protein interactions [10], together forming an integrated lactylation-centered regulatory network across physiological and pathological contexts.

Of particular note, the regulatory effects of lactate and lactylation are not restricted to metabolic enzymes or pathways, but extend to a broader oncogenic signaling network. Current research suggests that lactate and lactylation are associated with other oncogenes, such as Bcl-2 [39], FOS [40], Ras [41, 42], KIF23 [43] and so on, which have been shown to promote or induce cancer cell invasion and metastatic potential.

### Lactylation and lactate in the regulation of signaling pathways

Lactylation reflects metabolic alterations and exerts regulatory effects on gene expression, whereas lactate primarily functions as a signaling molecule that modulates key signaling pathways, together influencing tumor growth, metabolism, invasion, metastatic potential, and cancer progression.

The Hippo signaling pathway is known as a tumor suppressor mechanism, as it regulates the degradation of downstream components such as TAZ/YAP, but recent findings suggest that TAZ/YAP can promote tumor metabolism by supporting glycolysis [44]. In bladder cancer, circXRN2 overexpression stabilizes the upstream factor LATS1, leading to the activation of the Hippo pathway. As a result, enhanced degradation of TAZ/YAP suppresses glycolysis, leading to reduced lactate production and a subsequent decrease in H3K18 lactylation [8]. This axis illustrates how tumor-suppressive signaling pathways can indirectly reshape the lactylation landscape by modulating glycolytic activity and lactate availability, thereby linking Hippo pathway activation to epigenetic consequences downstream of metabolic reprogramming.

Following the discovery of the lactate receptor GPR81 on adipocyte membranes, lactate has been proposed to function as a signaling molecule [45]. Studies have revealed that GPR81 is expressed in various cell types, including muscle cells, immune cells, tumor cells, and cells of the central nervous system [46]. In this context, the biological effects are driven by lactate itself acting as an extracellular metabolite and ligand. Lactate, by binding to GPR81, is capable of modulating several signaling pathways, including the PI3K/AKT/CREB pathway [47], PI3K/AKT/mTOR pathway [48], mTOR/HIF-1α/STAT3 [49], Snail/EZH2/STAT3 pathway [50], HIF-1α pathway [51], Notch pathway [52] and Hedgehog pathway [53], thereby influencing processes such as tumor cell proliferation, angiogenesis, immune evasion, and metabolic reprogramming. Besides, through GPR81-mediated signaling, lactate stimulation induces the expression of PD-L1 in lung cancer cells, which is mediated by GPR81 [54]. Findings from research suggest that lactate-induced PD-L1 expression may inhibit T cell function, thus promoting tumor immune evasion [55]. Similarly, the interaction between lactate and GPR81 directly promotes the upregulation of BRCA1 [56] and ABCB [57], contributing to increased chemoresistance in tumors.

In addition, lactate is found to act as a signaling molecule to activate the NF-κB/IL-8 pathway, promoting tumor angiogenesis. Researches have demonstrated that lactate can be transported into endothelial cells via the monocarboxylate transporter MCT-1, where it induces the phosphorylation and degradation of IκBα, subsequently activating an autocrine NF-κB/IL-8 (CXCL8) signaling pathway that promotes cell migration and tube formation [58]. Inhibition of this pathway finds to be capable of reducing tumor invasiveness and metastatic potential.

Overall, lactate predominantly regulates tumor-related signaling pathways by acting as a metabolic signaling molecule, while lactylation represents a downstream epigenetic outcome of altered glycolytic flux and lactate availability. These mechanisms coordinate metabolic reprogramming, oncogenic signaling, and tumor progression, highlighting lactate’s central role in cancer biology.

## Uncovering tumor progressive and metastasis: lactate and lactylation disrupt cell adhesion, promote protein degradation, and drive angiogenesis

### Remodeling of cell adhesion by lactate

Accumulating evidence suggests that lactate directly mediates the remodeling of cell–extracellular matrix (ECM) adhesion. At the cellular level, such lactate-driven remodeling of adhesion is largely mediated through integrin-dependent interactions between tumor cells and the ECM.

Integrins are major adhesion molecules in the ECM that mediate various cellular functions in the body [59]. In tumor angiogenesis, carcinomas often exploit the overexpression of αv integrins to compete for vascular and stromal resources in order to encourage tumor progression and malignancy. In this regard, integrin αvβ3 integrin is indispensable for angiogenesis driven by the bFGF and TNF-α signaling pathways [60]. Research has shown that lactate enhances glioblastoma migration by modulating TGF-β2-induced expression of integrin αvβ3, while silencing LDH-A with siRNA effectively inhibits TGF-β2-driven tumor cell migration [61]. On top of that, acidification of the local TME, which is largely driven by excessive lactate production and proton export during enhanced glycolysis, reduces the binding affinity of integrins to the ECM and downregulates E-cadherin expression. Although this acidification weakens tumor cell adhesion to neighboring cells, it does not imply a reduced ability to invade surrounding vasculature or tissues; rather, a wealth of evidence shows that the acidic TME actually promotes invasive and metastatic phenotypes despite lower intercellular adhesion [62]. Accumulating evidence further indicates that lactic acid–induced acidification of the tumor microenvironment not only enhances local invasion but also increases vascular permeability [63], thereby facilitating cancer cell intravasation and entry into the blood circulation as part of the metastatic cascade. The dissolution of cell-cell contacts can facilitate single-cell detachment [64], enhance metalloproteinase activity and cytoskeletal reorganization, and support EMT and motility programs while extracellular acidosis concomitantly remodels stromal and endothelial compartments, rendering blood vessels more permissive to tumor cell intra- and extravasation. Together, these processes converge to promote increased invasion and intravasation.

### The cooperation of matrix metalloproteinase and lactate in protein degradation

ECM is crucial for maintaining cellular support and homeostasis, and its involvement is pivotal in the progression of cancer. Rapid tumor proliferation and enhanced glycolysis generate excessive lactate, which, together with the concomitant export of protons (H⁺), contributes to extracellular acidification in the tumor microenvironment [65]—a phenomenon recognized as a hallmark of cancer and characterized by a reversed pH gradient between intracellular and extracellular compartments [66]. Extracellular acidification is closely associated with lactate accumulation, and additional metabolic processes also participate in shaping tumor acidity. This lactate-associated acidic milieu stimulates the formation of new actin filaments, which, in turn, facilitates the binding of integrins on the cancer cell surface to ECM components, aiding in cell migration. Moreover, extracellular acidification resulting from tumor-associated metabolic alterations not only increases the number and size of tumor cell invadopodia but is also tightly coupled to the metabolic supply required to sustain their formation and activity. Acidic extracellular pH, driven by enhanced glycolysis and lactate export, promotes the recruitment and activation of the Na⁺/H⁺ exchanger NHE1 at invadopodia, leading to localized intracellular alkalinization. Invadopodia formation and function are energy-intensive processes that require localized ATP production to fuel actin polymerization, membrane trafficking, and protease activity at the protrusive site [67]. Glycolytic enzymes are enriched at invadopodia and support localized energy production through glycolysis [68], providing ATP in situ to maintain high actin dynamics and invadopodial ECM degradation activity. Disruption of glycolysis or ATP supply has been shown to impair invadopodia formation and ECM proteolysis [69], indicating that tumor metabolic reprogramming (e.g., Warburg-type glycolysis) directly fuels the energy demand of invadopodia. This pH gradient facilitates cofilin-dependent actin polymerization and Arp2/3-mediated cytoskeletal remodeling, processes that are energetically demanding and largely supported by ATP generated through high glycolytic flux. Thus, glycolysis-derived energy production and pH regulation are spatially coordinated at invadopodia to fuel actin dynamics, matrix degradation, and amoeboid tumor cell invasion, thereby reinforcing tumor progression [70]. This spatial coupling of glycolytic energy production and pH regulation highlights invadopodia as metabolically specialized subcellular domains that integrate bioenergetic supply with invasive function. On the other hand, alkalinization of the TME directly inhibits tumor invasion, highlighting the facilitating role of lactate-driven acidification for local tissue infiltration. TME acidification also activates proteinases, such as matrix metalloproteinase-9 (MMP-9), cathepsin B, and hyaluronidase-2, secreted by tumor cells, which degrade the surrounding matrix and further promote tumor cell invasion [71, 72]. The degradation of the ECM by secreted proteinases can facilitate the retention of growth factors like VEGF, TGF-β, and FGF2 within the matrix, thereby promoting tumor progression and angiogenesis [73]. Additionally, Lactate enhances glioma cell migration by upregulating the expression of transforming growth factor-beta 2 (TGF-β2), which subsequently stimulates the production of matrix metalloproteinase-2 (MMP-2) [61].

### The covert mechanism of tumor angiogenesis: lactate, lactylation and blood supply interplay

During tumor angiogenesis, lactate serves as a key metabolic signal that initiates pro-angiogenic pathways, whereas lactylation links sustained lactate accumulation to transcriptional reprogramming. Lactate promotes angiogenesis via stabilizing HIF-1α through both HIF-1α-dependent and -independent mechanisms [74]. In a prostate cancer study, it is observed that lactate could promote the upregulation of the HA-binding protein KIAA1199 through HIF1α-mediated Kla. KIAA1199 expression shows a positive correlation with tumor stage, HIF-1α overexpression, and angiogenic markers, suggesting that histone lactylation facilitates angiogenesis by enhancing gene transcription downstream of lactate signaling [9]. Notably, HIF-1α is a transcriptional enhancer of Vascular Endothelial Growth Factor A (VEGFA), which serves as the primary driver of angiogenesis. Lactate accumulation, together with lactylation as a downstream transcriptional outcome, contributes to angiogenesis through HIF-1α–VEGFA axis [75]. Lactate is involved in VEGF induction through the HIF-1α signaling pathway [51] while it can promote angiogenesis through a HIF-1α-independent mechanism as well. Lactate directly interacts with N-Myc downstream regulated gene 3 (NDRG3), a downstream regulator of N-Myc, preventing its degradation by PHD2/VHL and facilitating its integration with c-Raf. This interaction activates the Raf-ERK signaling pathway further driving angiogenesis, even if in hypoxic conditions with elevated lactate levels [46]. In clear cell renal cell carcinoma, the loss of VHL even under normal oxygen conditions enhances HIF activity, which contributes to the upregulation of glycolytic pathways and lactate production [76]. High lactate concentrations trigger histone H3K18 lactylation at the platelet-derived growth factor receptor β (PDGFRβ) promoter region in clear cell renal cell carcinoma. Lactylation also leads to excessive activation and transcription of PDGFRβ, causing excessive endothelial cell proliferation and abnormal vascular development. The PDGFRβ signaling can further promote histone lactylation, thereby establishing an oncogenic positive feedback loop [77]. In this loop, lactate-driven signaling initiates angiogenic activation, while lactylation reinforces and sustains the transcriptional program. Briefly, Lactate enhances angiogenesis by engaging multiple signaling pathways, while lactylation reinforces these angiogenic programs by amplifying endothelial proliferation and aberrant vascular remodeling at the epigenetic level. Lactate, acting as a signaling molecule, binds to GPR81 and subsequently modulates signaling pathways, triggering a series of biological effects. In breast cancer, elevated levels of GPR81 enhance cellular proliferation and stimulate angiogenesis through a mechanism dependent on the PI3K/AKT/CREB signaling pathway [47, 78].

In contrast to lactate-driven signaling, lactylation represents a downstream epigenetic adaptation to hypoxia and metabolic stress, which may influence tumor angiogenesis and therapeutic resistance. Inhibiting lactylation modifications in tumors may suppress tumor-driven angiogenesis given that the induction of hypoxia and the tumor’s response to it are crucial factors that influence the effectiveness of antiangiogenic therapy. Studies have illustrated that treatment with bevacizumab, an anti-angiogenic monoclonal antibody, causes elevated histone lactylation levels in drug-resistant metastatic colorectal cancer patients. Inhibition of histone lactylation effectively suppressed tumorigenesis, progression, and survival of metastatic colorectal cancer under hypoxic conditions. Histone lactylation promoted the transcription of RUBCNL/Pacer by interacting with BECN1 (beclin 1), mediating the recruitment and function of the class III phosphoinositide 3-kinase complex, which is of great significance in the proliferation and survival of hypoxic cancer cells [2]. Moreover, it is disclosed that the BMAL1 silencing-mediated LDHA/lactate axis promotes M1/M2 polarization, thereby enhancing the sensitivity of glioblastoma cells to bevacizumab and improving therapeutic efficacy [79]. Taken together, lactate acts as the metabolic driver of angiogenic signaling, and changes in lactylation reflect hypoxia-adaptive epigenetic programs that sustain tumor vascularization.

## How lactate and lactylation impacts on the “vitality” of cancer stem cells

Cancer stem cells (CSCs), a minor population within tumors marked by their ability to self-renew and proliferate indefinitely, are crucial contributors to tumor initiation, metastasis, and the preservation of tumor heterogeneity [80]. Stemness is defined as the characteristic of CSCs, which includes their abilities for self-renewal, resistance to apoptosis, and multipotent differentiation. Enhancing stemness often refers to strengthening these properties, hence increasing the role of CSCs in driving tumor progression [81]. Some scholars have proposed that CSCs may be the fundamental cause of tumor metastasis, suggesting that CSCs act as the ‘seed’ cells for tumor metastasis. Currently, growing attention has been directed toward the role of lactate metabolism and lactylation in the stemness of CSCs.

To begin with, research has revealed that lactate produced by LDHA in breast cancer modulates pH levels, facilitating USP28-mediated MYC deubiquitination and stabilization, which subsequently activates the SLUG promoter and drives the formation of breast cancer stem cell-like traits [82].

Following that, lactate contributes to the maintenance of CSC stemness across various tumor types by modulating metabolic pathways. For instance, the metabolic pathway of D-lactate promotes its clearance and pyruvate accumulation through the CDK7-YAP-LDHD axis, enabling esophageal squamous cell carcinoma (ESCC) CSCs to evade ferroptosis. This process also fulfills the energy demands necessary for their self-renewal potential, thereby sustaining their stemness properties [83].

In addition, elevated glycolytic metabolism, lactate accumulation, and increased levels of lactylation in liver CSCs are strongly linked to tumorigenesis and the maintenance of stemness. Notably, H3K56la and lactylation of ALDOA at K230/322 play a critical role in enhancing the stemness of liver CSCs by influencing the interaction between ALDOA and DDX17, thus regulating the expression of stemness-associated genes [6].

Studies have also demonstrated that lactate treatment increases intracellular lactate levels, subsequently promoting histone H3 lactylation, particularly at K9, K14, K23, and K56 residues. This ultimately brings about enhanced proliferation and viability of liver CSCs. The lactylation of these sites may be implicated in the onset and progression of liver cancer [84].

In summary, lactate metabolism and lactylation regulate CSCs stemness through various mechanisms. Owing to the inhibition of CSCs which can serve as an effective therapeutic strategy to suppress metastasis and invasion, deeper exploration of these mechanisms will provide new targets and strategies for anti-tumor therapy.

## Lactate and lactylation in the TME: a key modulator of immune evasion

**Fig. 3:**
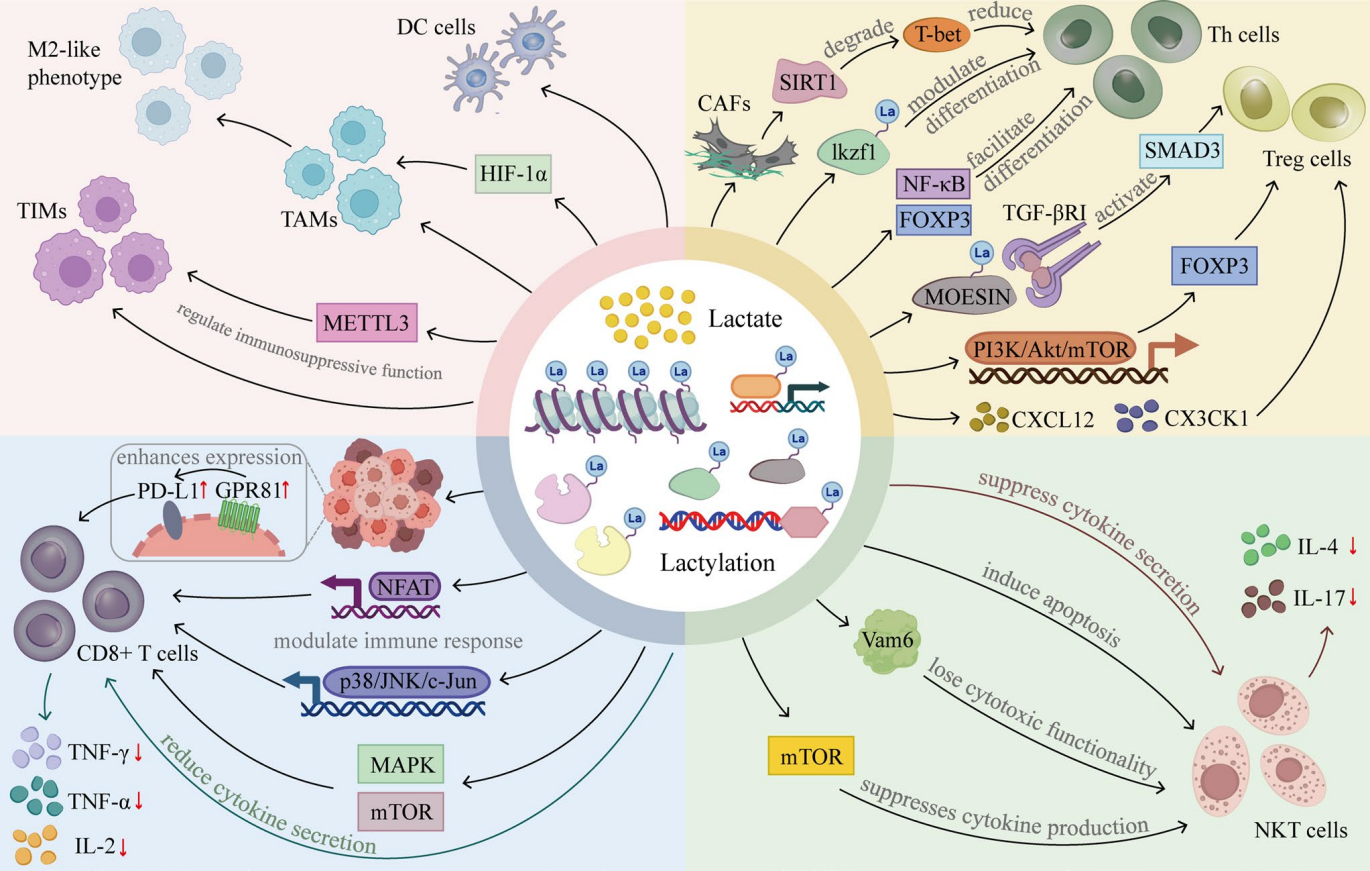
The main mechanisms of lactate and lactylation on immune cells. Lactate and lactylation influence different immune cells through several critical mechanisms, thereby facilitating tumor immune escape and promoting tumor progression and metastasis

Mechanistically, lactate primarily functions as a metabolic substrate and signaling molecule that directly modulates immune cell activity, whereas lactylation represents a downstream epigenetic response to altered lactate metabolism, fine-tuning gene expression programs associated with immune cell differentiation and function. To provide an integrated overview of these multifaceted immunoregulatory effects, Fig. 3 summarizes the principal mechanisms by which lactate and lactylation modulate both innate and adaptive immune cells within the tumor microenvironment. As illustrated, lactate primarily acts as a metabolic substrate and signaling molecule, whereas lactylation functions as a downstream epigenetic mechanism that fine-tunes immune cell differentiation, effector function, and immunosuppressive activity. Through coordinated effects on tumor-infiltrating myeloid cells (TIMs), dendritic cells (DC cells), CD8⁺ T cells, CD4⁺ T helper cells (Th cells) and regulatory T cells (Treg cells) and natural killer T cells (NKT cells), the accumulation of lactate in the tumor microenvironment reflects a profound metabolic rewiring that not only supports tumor growth but also systematically reprograms immune cell function, thereby weakening immune surveillance and facilitating immune escape.

Importantly, the immunological consequences of lactate accumulation are not uniformly suppressive but are highly dependent on its concentration, spatial distribution, and cellular context. Under certain conditions, lactate has been reported to support pro-inflammatory signaling or effector functions, highlighting the need to interpret lactate-driven immune regulation in a dose- and microenvironment-specific manner. In lactate-rich but not uniformly suppressive microenvironments, lactate can reinforce pro-inflammatory immune programs. Sodium lactate, in particular, has been shown to promote IL-17 production in CD4⁺ T cells, establishing a lactate–IL-17 feed-forward loop that enhances inflammatory signaling and induces a stop-migration phenotype, thereby retaining T cells at inflamed sites [85, 86]. In parallel, lactate can stabilize hypoxia-inducible factor-1α (HIF-1α), a transcriptional regulator that favors Th17 differentiation while antagonizing Treg cell development, further biasing adaptive immunity toward inflammation [87]. By contrast, when lactate accumulation is accompanied by extracellular acidification, lactic acid suppresses CD8⁺ T cell motility and cytotoxic function, underscoring the importance of proton availability as a key contextual determinant of lactate’s immune impact [86]. Together, these findings indicate that lactate exerts context-, concentration-, and pH-dependent immunomodulatory effects within the tumor microenvironment.

### TIMs

TIMs, as major components of the innate immune system, play a critical role in shaping the immunosuppressive tumor microenvironment. In the TME, lactate regulates the immunosuppressive function of TIMs through the induction of METTL3 expression and lactylation modifications, revealing a novel mechanism of immune regulation [35].

Among TIMs, tumor-associated macrophages (TAMs) represent one of the most extensively studied immune populations in the context of tumor metabolism and immune evasion. Accumulating evidence from classical studies has demonstrated that tumor-derived lactate acts as a key metabolic cue driving TAM polarization toward an immunosuppressive, M2-like phenotype [1], thereby facilitating tumor progression, angiogenesis, and immune escape. These findings establish TAMs as a central immune population through which lactate links tumor metabolism to immune suppression. Lactate-enriched TMEs favor macrophage metabolic reprogramming and functional skewing, reinforcing their pro-tumorigenic activities.

Mechanistically, lactate has been shown to stabilize hypoxia-inducible factor-1α (HIF-1α) in TAMs, a central regulator of macrophage adaptation to the tumor microenvironment. Beyond metabolic signaling alone, recent studies have demonstrated that lactate induces histone H3 lysine 18 lactylation (H3K18la) at promoter and enhancer regions of macrophage polarization–associated genes, thereby directly shaping TAM transcriptional programs [88]. H3K18la is enriched at loci encoding immunosuppressive and pro-tumorigenic factors, including Arg1, IL-10, VEGFA, and other M2-associated genes, leading to their sustained transcriptional activation. This lactylation-dependent chromatin remodeling cooperates with HIF-1α–driven transcription, reinforcing M2-like polarization and stabilizing the immunosuppressive phenotype of TAMs even under fluctuating metabolic conditions. Through this epigenetic mechanism, lactate is not merely a metabolic byproduct but functions as a chromatin-modifying signal that imprints long-lasting transcriptional memory in TAMs, thereby consolidating macrophage-mediated immune suppression and tumor support within the TME.

### Lactate and adaptive immune cells

In addition to innate immunity, lactate exerts profound regulatory effects on adaptive immune responses, particularly T cell–mediated antitumor immunity.

#### CD8 + T cells

Lactate imposes suppressive effects on CD8 + T cells by impairing key aspects of their function. Consistent with this notion, lactate has been demonstrated to directly modulate T cell–driven immune responses, in part through the regulation of key signaling cascades, including the NFAT and p38/JNK/c-Jun pathways, which are essential for sustaining cytokine secretion and cytotoxic activity [89, 90].

Beyond signaling modulation, elevated lactate in the TME reshapes CD8⁺ T cell metabolism by disrupting glycolytic flux and redox balance, thereby limiting the bioenergetic and biosynthetic capacity required for effective antitumor responses. Tumor-derived lactate also perturbs key metabolic pathways such as MAPK and mTOR, which are integral to T cell activation and survival [91], and stimulates histone lactylation that correlates with exhaustion phenotypes in CD8⁺ T cells [92]. Given the tight coupling between metabolic fitness and effector function in CD8⁺ T cells — a relationship supported by studies showing that glycolytic flux is required for efficient IFN-γ production and other effector activities — such metabolic constraints directly translate into impaired cytokine production [93, 94]. As a consequence, lactate exposure leads to reduced production of key effector cytokines, including IFN-γ, TNF-α, and IL-2, which are indispensable for CD8⁺ T cell–mediated tumor control [89, 95].

In parallel, lactate facilitates tumor immune evasion by modulating immune regulatory ligands and receptors. Specifically, lactate facilitates tumor immune evasion by modulating the expression of immune regulatory ligands and receptors. To be specific, it enhances programmed death-ligand 1 (PD-L1) expression on tumor cells via its receptor GPR81, thus suppressing CD8 + T-cell cytotoxicity and fostering immune tolerance within the TME [54].

Emerging evidence further suggests that lactylation participates in the epigenetic regulation of CD8⁺ T cell function. Enrichment of H3K18la and H3K9la has been associated with transcriptional programs linked to CD8⁺ T cell exhaustion and functional impairment, indicating that lactylation may act as a downstream epigenetic mechanism reinforcing lactate-induced immunosuppression [96]. Integrated evidence indicates that lactate and its downstream lactylation regulate T cell cytotoxicity and cytokine secretion through both metabolic and epigenetic mechanisms, effectively linking metabolic reprogramming to immune dysfunction in cancer immunity [97].

In aggregate, these findings highlight that lactate suppresses CD8⁺ T cell–mediated antitumor immunity through coordinated metabolic, signaling, and epigenetic mechanisms, thereby contributing to the establishment of an immunosuppressive tumor microenvironment. Recent studies have begun to demonstrate that targeting lactate production/transport or modulating lactylation may restore CD8⁺ functionality, providing a therapeutic avenue to enhance immunotherapy responses [98].

#### CD4⁺ T cells and Treg cells

Recent findings have shed light on lactate’s regulatory influence over CD4 + T cell function within the TME. CAFs mediate a reduction in Th1 cell populations through a lactate-dependent mechanism involving the SIRT1-driven degradation of T-bet [99]. Simultaneously, this lactate-rich environment facilitates Treg differentiation via activation of the NF-κB and FOXP3 pathways. Moreover, sodium lactate has been reported to skew CD4⁺ T cell differentiation toward a proinflammatory Th17 phenotype, accompanied by impaired T cell motility resulting from glycolytic disruption [85]. Notably, T helper cells (TH17 cells) exhibit functional plasticity within the TME, where they may either support antitumor immunity or contribute to immune evasion depending on metabolic and epigenetic cues. Consistent with this plasticity, Th17 cells may exert either antitumor or protumor functions depending on lactate availability, metabolic state, and epigenetic regulation, rather than serving as a uniformly immunosuppressive population within the TME. In addition to metabolic regulation, lactylation has emerged as a key epigenetic mechanism shaping CD4⁺ T cell differentiation. Lactylation of Ikzf1 at Lys164 is elevated in CD4⁺ T cells and modulates Th17 differentiation by altering Ikzf1 binding to the promoters of Th17-associated genes, including Runx1, Tlr4, IL-2, and IL-4; mutation of this site disrupts Th17 lineage commitment [100]. On top of that, lactate-induced lactylation promotes metabolic and epigenetic reprogramming of Th17 cells, facilitating their phenotypic conversion toward Treg cells and thereby favoring immune suppression within the TME [101].

Treg cells play a central role in maintaining immune tolerance and suppressing antitumor immune responses. Lactate enhances Treg function by inducing lactylation of MOESIN at Lys72, which strengthens its interaction with TGF-β receptor I and activates downstream SMAD3 signaling, ultimately reinforcing FOXP3 expression and Treg suppressive capacity. Inhibition of MOESIN lactylation significantly attenuates Treg-mediated immunosuppression [102]. Accordingly, targeting lactate production through LDH inhibition reduces Treg induction, restores antitumor immunity, and markedly slows tumor growth. Importantly, the persistence of Treg cells within the tumor microenvironment is also supported by their intrinsic metabolic adaptability to acidic and nutrient-deprived conditions. In acidic and nutrient-limited microenvironments, Treg cells display intrinsic metabolic adaptability that supports their survival and baseline functional fitness, a process that has been linked to PI3K–mTOR signaling and metabolic substrate utilization [103]. The metabolic adaptability that enables Treg survival in acidic environments should be distinguished from lactate-induced epigenetic mechanisms that actively enhance suppressive function, underscoring mechanistic heterogeneity within lactate-driven immune regulation [104]. Beyond supporting Treg survival, lactate further promotes Treg accumulation and immunosuppressive dominance by activating PI3K/Akt/mTOR signaling and inducing chemokines such as CXCL12 and CX3CL1, reinforcing the immunosuppressive tumor milieu [105–107]. These findings indicate that Treg-mediated immunosuppression in the TME is sustained by a combination of lactate-driven epigenetic reinforcement of suppressive function and metabolic adaptations that permit their persistence and accumulation under hostile tumor conditions.

### NKT cells

NKT cells are particularly susceptible to the immunosuppressive effects of lactate. A high-lactate environment is detrimental to the survival and proliferation of NKT cells, inducing apoptosis, reducing their numbers, and impairing their anti-tumor immune function. Moreover, Elevated lactate levels suppress cytokine secretion, causing a decrease in IL-4 and IL-17 expression in NKT cells [108]. Lactate also suppresses cytokine production in NKT cells by inhibiting mTOR signaling, a pathway essential for their activation and effector function [109]. Additionally, lactate has been implicated in upregulating immunosuppressive molecules such as Vam6 in invariant NKT cells, which correlates with a loss of cytotoxic functionality [110]. Researchers have demonstrated that tumors exhibiting high lactylation scores, serving as a metric to assess the overall expression levels and functional activity of lactylation-related genes in tumor samples, show significantly reduced infiltration of NKT cells, suggesting that lactylation suppresses both the infiltration and functional activity of NKT cells by modulating immune regulatory mechanisms within the TME [111]. Through apoptosis induction, cytokine suppression, and enhanced expression of immunosuppressive molecules, Immune regulatory mechanisms in the modulated TME, lactate exacerbates the TME’s immunosuppressive effects, further compromising NKT cell-mediated anti-tumor responses.

In summary, lactate and lactylation cooperatively remodel the tumor immune landscape by reprogramming innate and adaptive immune cells. Through metabolic signaling and epigenetic adaptation, they facilitate immune evasion, promote tumor survival, and ultimately drive tumor progression and metastasis.

## Lactylation is an emerging target in tumor therapy and future challenges

**Fig. 4:**
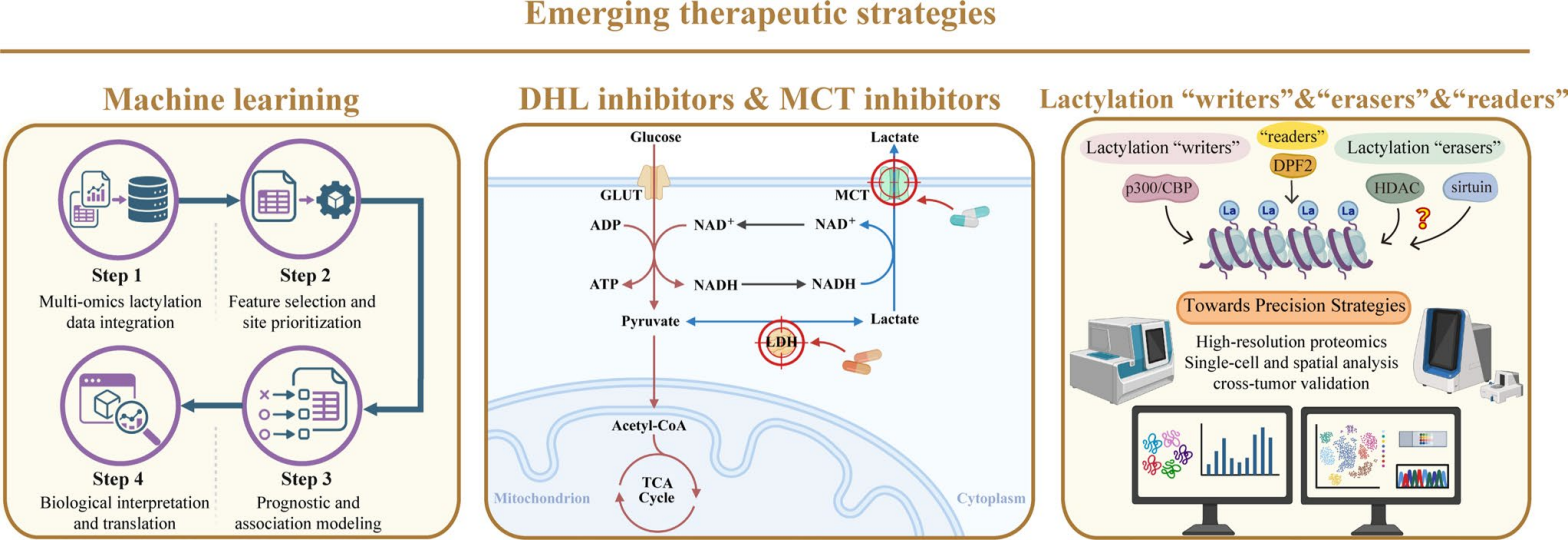
Emerging therapeutic targeting of lactate metabolism and lactylation in cancer

Emerging therapeutic strategies targeting lactate metabolism and lactylation in cancer encompass three interconnected layers—computational modeling–assisted target discovery, modulation of lactate production and transport via LDH and MCTs, and direct intervention in lactylation through its epigenetic “writers” and “erasers” (Fig. 4)—together providing a multidimensional framework to disrupt lactate-driven tumor progression.

Although recent basic research has identified numerous lactylation modification sites, the sheer scale, heterogeneity, and context-dependent nature of these modifications pose substantial analytical challenges. With the advancement of machine learning, computational approaches have therefore been introduced as complementary tools to address limitations inherent to conventional experimental and statistical analyses in cancer lactylation research. One major pain point lies in the difficulty of systematically prioritizing potentially functionally relevant lactylation sites from high-dimensional omics datasets, as experimental validation of each candidate site is labor-intensive and costly. By integrating large-scale gene expression profiles, proteomic data, and known PTM features, machine learning algorithms have been used to suggest lactylation sites that are most strongly associated, in specific datasets, with tumor development, metabolic reprogramming, and disease maintenance [3, 112]. However, it should be noted that most current studies rely on retrospective analyses with limited sample sizes, and external validation across independent cohorts remains variable. These data-driven prioritization strategies may provide a rational starting point for focusing experimental efforts and highlight lactylation sites with potential translational relevance, rather than definitive therapeutic targets. Another critical challenge is the limited ability of traditional models to capture nonlinear and multivariate relationships between lactylation patterns and clinical outcomes. Leveraging curated lactylation signatures, machine learning-based prognostic models have been proposed for multiple malignancies, including lymphoma [113], breast cancer [114], lung cancer [115], pancreatic cancer [116] and so on. Most of these models are developed using single-cancer cohorts and moderate sample sizes, and their generalizability across tumor types or clinical settings has not yet been systematically established. In terms of clinical operability, these models are currently best suited for exploratory risk stratification or hypothesis generation, rather than direct clinical decision-making. Their predictive performance may be most applicable in scenarios such as retrospective outcome prediction, patient subgroup stratification, or treatment response estimation under defined conditions, rather than prospective individualized therapy guidance. In combination, machine learning should not be viewed as a standalone predictive solution, but rather as a hypothesis-generating framework that may facilitate the functional interpretation of complex lactylation landscapes and potentially accelerate the translation of lactylation research toward precision oncology.

LDH mediates the bidirectional conversion between pyruvate and lactate and is an emerging anticancer target. Elevated levels of LDHA serve as a hallmark in malignant tumor such as glioblastoma [117], breast cancer [4], gastric cancer [7], cervical cancer [118] and so on, most of which are highly dependent on glycolysis. Given that high expression of LDHA in numerous tumors has been associated with impairing immune response, disabled immune surveillance and poor prognosis, several LDHA inhibitors are under development [119].

MCTs, responsible for the transport of lactate across cell membranes, are of great significance in the import and export of lactate, particularly in cells with high glycolytic activity. Specifically, the increased expression of MCT1 and MCT4 in cancer has been observed across multiple tumor types, creating opportunities for therapeutic intervention, allowing for the potential inhibition of these transporters through small-molecule inhibitors or by targeting their cochaperone CD147, such as with CD147-specific antibody therapies [120, 121]. Meanwhile, MCT inhibitors are being actively developed, with small-molecule inhibitors currently undergoing clinical trials.

Beyond targeting lactate production and transport, increasing attention has been directed toward the therapeutic feasibility of intervening in lactylation itself, particularly through modulation of lactylation “writers” and “erasers”. Emerging evidence suggests that the histone acetyltransferase p300 exhibits broad acyltransferase promiscuity and can utilize multiple acyl-CoA substrates, thereby catalyzing various histone lysine acylations in response to intracellular metabolic states. In this context, p300 has been proposed to function as a candidate lactylation writer, although whether p300/CBP possesses bona fide lactyltransferase activity in vivo remains controversial [1, 122]. Pharmacological inhibition of p300 may therefore suppress aberrant histone acylation programs driven by elevated lactate or altered acyl-CoA pools, potentially attenuating tumor-promoting transcriptional outputs. Conversely, the identity of lactylation “erasers” is considerably less well defined. Histone deacetylases (HDACs), traditionally recognized as Zn²⁺-dependent deacetylases, exhibit limited capacity to remove non-acetyl acyl modifications. In humans, the classical Zn²⁺-dependent HDAC family comprises multiple members that mediate deacetylation: class I HDACs (HDAC1, HDAC2, HDAC3 and HDAC8) are predominant histone deacetylases with strong activity toward histone lysine acetylation, class IIb members such as HDAC6 and HDAC10 also display deacetylase activity often directed toward non-histone substrates, and class IV contains HDAC11 with deacetylase function [123, 124]. According to biochemical profiling studies, most Zn²⁺-dependent HDACs display negligible activity toward short-chain lysine acylations, with HDAC3 showing only weak decrotonylase activity in vitro, and no definitive evidence supporting robust delactylation activity [125]. By comparison, members of the sirtuin family have been shown to possess broader deacylase activities, raising the possibility that lactylation removal may preferentially depend on NAD⁺-dependent enzymes rather than classical HDACs; however, direct and site-specific delactylation by individual sirtuins has yet to be systematically established.

Unlike LDHA or MCT inhibition, which globally alters lactate availability, targeting lactylation writers or erasers may enable a more selective disruption of lactate-driven epigenetic reprogramming without completely abolishing the physiological metabolic functions of lactate. However, the translational potential of lactylation-targeted strategies is currently constrained by both conceptual and technical limitations. From a mechanistic perspective, the promiscuity of candidate writers, the incomplete identification of bona fide erasers, and the absence of clearly defined lactylation “readers” complicate efforts to precisely manipulate this modification without perturbing broader epigenetic networks. Although a few studies have begun to nominate potential lactylation readers—such as DPF2 [126] binding to H3K14la or Brg1 [127] recognizing H3K18la in specific cellular contexts—these interactions have so far been demonstrated in limited experimental systems and appear to be highly context-dependent, with unclear binding specificity, stoichiometry, and functional generalizability. The unresolved upstream regulatory features underscore the need for rigorous biochemical validation and context-specific functional interrogation.

From a methodological standpoint, the accurate detection and quantification of lactylation remain challenging, as lactylation marks are often low-abundance, highly dynamic, and susceptible to confounding by structurally related lysine acylations in mass spectrometry–based analyses. Moreover, most existing datasets are derived from bulk tumor samples or limited experimental models, which may obscure cell-type–specific lactylation landscapes within the tumor microenvironment. At the biological level, profound metabolic heterogeneity across tumor types, disease stages, and microenvironmental contexts leads to substantial variability in lactate availability and lactylation dependency. Consequently, mechanisms identified in highly glycolytic tumors may not be universally applicable, underscoring the need for context-aware interpretation. Addressing these challenges will require advances in high-resolution proteomics, single-cell or spatially resolved analyses, and systematic cross-tumor comparisons, thereby providing a more robust foundation for the rational development of lactylation-targeted therapeutic strategies.

## Conclusion

Lactate and lactylation represent vital players in shaping the TME and driving cancer progression. The impact of lactate and lactylation on tumor biology is multifaceted and extend beyond simple metabolic byproducts, as they actively participate in reprogramming cell signaling networks, disrupting cell adhesion, enhancing protein degradation, promoting angiogenesis and influencing CSCs. Essentially, lactate-driven mechanisms also support immune escape, contributing to the suppression of anti-tumor immunity. Understanding how lactate and lactylation regulate these diverse cellular functions offers promising avenues for therapeutic interventions. Targeting lactate metabolism and its associated modifications could provide novel strategies for overcoming treatment resistance and improving cancer therapy outcomes. Future researches are supposed to focus on elucidating the detailed molecular mechanisms underlying lactate and lactylation’s multifaceted effects, enabling the development of targeted therapies that can exploit these metabolic and epigenetic alterations to combat cancer more effectively.

## Data Availability

No datasets were generated or analysed during the current study.

## Abbreviations

TME: Tumor Microenvironment
Kla: Lysine Lactylation
PTM: Post-translational Modification
CAFs: Cancer-associated Fibroblasts
NSCLC: Non-small Cell Lung Cancer
ECM: Extracellular Matrix
CSCs: Cancer Stem Cells
ESCC: Squamous Cell Carcinoma
EMT: Epithelial-Mesenchymal Transition
Treg: Cells Regulatory T cells
NKT: Cells Natural Killer T cells
TIMs: Tumor-infiltrating Myeloid Cells
TAMs: Tumor-associated Macrophages
MCTs: Monocarboxylate Transporters
PD-L1: Programmed Death-Ligand 1
HDACs: Histone Deacetylases
H3K18la: Histone H3 Lysine 18 Lactylation
